# Supplementary material for: Variational quantum generative modeling by sampling expectation values of tunable observables
Source: npj Quantum Inf. 2025 Nov 18;11(1):178. doi: 10.1038/s41534-025-01121-x (PMC12626880; doi:10.1038/s41534-025-01121-x)
Supplement: Supplementary file 1 — Supplementary information [file 41534_2025_1121_MOESM1_ESM.pdf]

Supplementary Information for Variational Quantum Generative Modeling by Sampling Expectation Values of Tunable Observables

Kevin Shen,[1,2,3,*] Andrii Kurkin,[1,2,3] Adrián Pérez-Salinas,[1,4]
Elvira Shishenina,[3,†] Vedran Dunjko,[1,2] and Hao Wang[1,2]

[1] $\langle aQa^L \rangle$ Applied Quantum Algorithms, Leiden University, The Netherlands
[2] LIACS, Leiden University, Niels Bohrweg 1, 2333 CA, Leiden, The Netherlands
[3] BMW Group, 80788 München, Germany
[4] Instituut-Lorentz, Leiden University, Niels Bohrweg 2, 2333 CA, The Netherlands

SUPPLEMENTARY NOTE 1 – MODEL EXPRESSIVITY

We extend the main text's discussion on the expressivity of the Observable-Tunable Expectation Value Sampler (OT-EVS). We verify the validity of the two examples given in the main text.

In Example 1, outputs of the OF-EVS take the form:

$$y_m = \sin\left(\frac{\theta_m + z_m}{2}\right) \quad \text{for } m \in \{1, 2\}, \tag{1}$$

while outputs of the OT-EVS take the form $(\boldsymbol{\alpha}_{11}y_1 + \boldsymbol{\alpha}_{12}y_2, \boldsymbol{\alpha}_{21}y_1 + \boldsymbol{\alpha}_{22}y_2)$. We can attribute the increase in expressivity to two causes. First, linear combinations of y_1 and y_2 follow different distributions than y_1 or y_2 in general, even though y_1 and y_2 are identically distributed up to $\boldsymbol{\theta}$. Second, $\boldsymbol{\alpha}_{11}y_1 + \boldsymbol{\alpha}_{12}y_2$ is in general correlated with $\boldsymbol{\alpha}_{21}y_1 + \boldsymbol{\alpha}_{22}y_2$, while y_1 and y_2 themselves are always independent.

To verify Example 2, we utilize existing theoretical tools [1]. When measuring a Haar-random state, the expectation value of a given observable O is a random variable derived from the inner product of a symmetric Dirichlet random variable and the eigenvalues of the observable. Consider an observable $O_{\boldsymbol{\alpha}}$ defined by weighted sums of O and some other observables. The eigenvalues of $O_{\boldsymbol{\alpha}}$ will depend on the spectra of all constituent observables and their weights. Consequently, the resulting spectrum and the underlying distribution of the expectation value differ from those of O originally.

SUPPLEMENTARY NOTE 2 – PROOF OF THEOREM 1 OF THE MAIN TEXT

First, we denote by $\boldsymbol{y}^i$ and $\tilde{\boldsymbol{y}}^i$ the i-th sample (in a batch) generated by the shot-noise-free and shot-noise perturbed OT-EVS in shadow-frugal parameterization, respectively:

$$\boldsymbol{y}^i = \left[\mathrm{tr}\big(H_1\rho^i\big), \dots, \mathrm{tr}\big(H_M\rho^i\big)\right]^\top = \left[\sum_{l=1}^{L} \boldsymbol{\alpha}_{1l}\langle O_l^i\rangle, \cdots, \sum_{l=1}^{L} \boldsymbol{\alpha}_{Ml}\langle O_l^i\rangle\right]^\top = \boldsymbol{\alpha}\left[\langle O_1^i\rangle, \cdots, \langle O_L^i\rangle\right]^\top = \boldsymbol{\alpha}\langle \boldsymbol{O}^i\rangle. \tag{2}$$

$$\tilde{\boldsymbol{y}}^i = \left[\hat{h}_1^i, \dots, \hat{h}_M^i\right]^\top = \left[\sum_{l=1}^{L} \boldsymbol{\alpha}_{1l}\widehat{\langle O_l^i\rangle}, \cdots, \sum_{l=1}^{L} \boldsymbol{\alpha}_{Ml}\widehat{\langle O_l^i\rangle}\right]^\top = \boldsymbol{\alpha}\left[\widehat{\langle O_1^i\rangle}, \cdots, \widehat{\langle O_L^i\rangle}\right]^\top = \boldsymbol{\alpha}\widehat{\langle \boldsymbol{O}^i\rangle}, \tag{3}$$

where $\widehat{\langle O_l^i\rangle}$ is the estimation of the expectation value of the l-th Pauli string for the i-th sample by a particular measurement strategy. Next, we prove two lemmas required for Theorem 1.

Lemma 1. *Let L denote the number of k-local Pauli strings, and let B represent the batch size. Denote $\mathbb{P}^B$ and $\tilde{\mathbb{P}}^B$ as the empirical distributions of the shot-noise-free and shot-noise-perturbed samples, respectively, generated by an OT-EVS under the shadow-frugal parameterization specified by a weight matrix $\boldsymbol{\alpha}$ and let $\|\boldsymbol{\alpha}\|_\infty \le T$. Then, for any*

---

* kevin.shen@bmwgroup.com
† Now at Quantinuum, Leopoldstrasse 180, 80804 München, Germany.

$\epsilon > 0$, the following inequality can be established for the $\mathcal{W}_1$-distance between the shot-noise-free distribution $\mathbb{P}^B$ and the shot-noise-perturbed distribution $\tilde{\mathbb{P}}^B$:

$$\Pr(\mathcal{W}_1(\mathbb{P}^B, \tilde{\mathbb{P}}^B) \geq \epsilon) \leq \Pr\left(\bigcup_{i=1}^{N}\bigcup_{l=1}^{L}\left\{|\langle O_l^i\rangle - \widehat{\langle O_l^i\rangle}| \geq \frac{\epsilon}{T}\right\}\right). \tag{4}$$

Proof. Using definition of $\mathcal{W}_1$-distance and properties of the norm we have:

$$\mathcal{W}_1(^B, \tilde{\mathbb{P}}^B) = \inf_{\pi \in S_N}\left(\frac{1}{N}\sum_{i=1}^{N}\|\boldsymbol{y}^i - \tilde{\boldsymbol{y}}^{\pi(i)}\|_1\right) \leq \frac{1}{N}\sum_{i=1}^{N}\|\boldsymbol{y}^i - \tilde{\boldsymbol{y}}^i\|_1 \tag{5}$$

$$= \frac{1}{N}\sum_{i=1}^{N}\|\boldsymbol{\alpha}(\langle \boldsymbol{O}^i\rangle - \widehat{\langle \boldsymbol{O}^i\rangle})\|_1 \leq \frac{1}{N}\sum_{i=1}^{N}\|\boldsymbol{\alpha}\|_\infty\|\langle \boldsymbol{O}^i\rangle - \widehat{\langle \boldsymbol{O}^i\rangle}\|_\infty \tag{6}$$

$$\leq \frac{T}{N}\sum_{i=1}^{N}\max_l|\langle O_l^i\rangle - \widehat{\langle O_l^i\rangle}|. \tag{7}$$

Taking into account the probability inequality:

$$\Pr(\mathcal{W}_1(\mathbb{P}^B, \tilde{\mathbb{P}}^B) \geq \epsilon) \leq \Pr\left(\frac{1}{N}\sum_{i=1}^{N}\max_l|\langle O_l^i\rangle - \widehat{\langle O_l^i\rangle}| \geq \frac{\epsilon}{T}\right) \leq \Pr\left(\bigcup_{i=1}^{N}\bigcup_{l=1}^{L}\left\{|\langle O_l^i\rangle - \widehat{\langle O_l^i\rangle}| \geq \frac{\epsilon}{T}\right\}\right). \tag{8}$$

□

Lemma 2 (restatement of Th1, p.13 and L3, p.26 from [2])**.** *Fix a measurement primitive $\mathcal{U}$ of randomly chosen Pauli measurements, a collection of observables $P_1, \ldots, P_L$, which are at most k-local Pauli strings. Fix accuracy parameters $\epsilon, \delta \in [0, 1]$. Then, a collection of*

$$K = \left\lceil 68\frac{3^k}{\epsilon^2}\log\left(\frac{2L}{\delta}\right)\right\rceil \tag{9}$$

independent classical shadows allow for accurately predicting expectation values of all observables via the median of means prediction, which means that:

$$|\hat{p}_l(K) - \mathrm{tr}\,(P_l\rho)| \leq \epsilon \quad \textit{for all } 1 \leq l \leq L \tag{10}$$

with probability at least $1 - \delta$.

Next, we show the main theorem again and prove it with the above lemmas.

Theorem 1 (Sample Complexity)**.** *Let L be the number of k-local Pauli strings. Let B be the batch size. Denote by $\mathbb{P}^B$ the empirical noiseless distribution of a batch of samples generated from an OT-EVS with shadow-frugal parameterization, and by $\tilde{\mathbb{P}}^B$ the distribution with measurement error. We assume $\|\boldsymbol{\alpha}\|_\infty \leq T$, implying $\|A_m\|_\infty \leq T$, $\forall 1 \leq m \leq M$, where $A_m = \sum_{l=1}^{L}\boldsymbol{\alpha}_{m,l}O_l$. For $\epsilon, \delta > 0$, the following probabilistic bound*

$$\Pr\left(\mathcal{W}_1(\mathbb{P}^B, \tilde{\mathbb{P}}^B) \leq \epsilon\right) > 1 - \delta \tag{11}$$

holds with measurements N_s,

$$N_s \geq 68 \cdot 3^k\left\lceil B\frac{T^2}{\epsilon^2}\log\left(\frac{2LB}{\delta}\right)\right\rceil \; \textit{(with classical shadows)} \tag{12}$$

$$N_s \geq 2L\left\lceil B\frac{T^2}{\epsilon^2}\log\left(\frac{2LB}{\delta}\right)\right\rceil \textit{(with the conventional measurements)} \tag{13}$$

Proof. Conventional Measurements: Using Lemma 1 and Boole's inequality, we can place an upper bound on the probability for the expression involving the $\mathcal{W}_1$-distance between distributions and then impose a condition that this probability is sufficiently small:

$$\Pr(\mathcal{W}_1(\mathbb{P}^B, \tilde{\mathbb{P}}^B) \geq \epsilon) \leq \Pr\left(\bigcup_{i=1}^{B}\bigcup_{l=1}^{L}\left\{|\langle O_l^i\rangle - \widehat{\langle O_l^i\rangle}| \geq \frac{\epsilon}{T}\right\}\right) \leq \sum_{i=1}^{B}\sum_{l=1}^{L}\Pr\left(\left|\hat{o}_l^i(K) - \mathrm{tr}\left(O_l\rho^i\right)\right| \geq \frac{\epsilon}{T}\right) \leq \delta. \tag{14}$$

For any fixed $i \in [B]$ and $l \in [L]$ we compute average over K measurements of ρ_i, where K is chosen such that:

$$\left|\hat{o}_l^i(K) - \operatorname{tr}\left(O_l \rho^i\right)\right| \leq \frac{\epsilon}{T} \quad \text{for all } 1 \leq i \leq B,\, 1 \leq l \leq L \tag{15}$$

with failure probability $\frac{\delta}{BL}$. Using Hoeffding inequality, we can compute K:

$$\Pr\left(\left|\hat{o}_l^i(K) - \operatorname{tr}\left(O_l \rho^i\right)\right| \geq \frac{\epsilon}{T}\right) \leq 2\exp\left\{-\frac{2K^2\epsilon^2}{4KT^2}\right\} \leq \frac{\delta}{BL}. \tag{16}$$

Thus, $K = \left\lceil 2\frac{T^2}{\epsilon^2}\log\left(\frac{2BL}{\delta}\right)\right\rceil$. Multiplying it by BL gives us the total number of measurements.

Classical Shadows: Using Lemma 1 and Boole's inequality, we can place an upper bound on the probability for the expression involving the $\mathcal{W}_1$-distance between distributions and then impose a condition that this probability is sufficiently small:

$$\Pr(\mathcal{W}_1(\mathbb{P}^B, \tilde{\mathbb{P}}^B) \geq \epsilon) \leq \Pr\left(\bigcup_{i=1}^{B}\bigcup_{l=1}^{L}\left\{|\langle O_l^i\rangle - \widehat{\langle O_l^i\rangle}| \geq \frac{\epsilon}{T}\right\}\right) \leq \sum_{i=1}^{B}\Pr\left(\bigcup_{l=1}^{L}\left\{\left|\hat{o}_l^i(K) - \operatorname{tr}\left(O_l^i\rho^i\right)\right| \geq \frac{\epsilon}{T}\right\}\right) \leq \delta \tag{17}$$

For any fixed $i \in [B]$, the number of measurements K for constructing shadow array is chosen such that:

$$|\hat{o}_l(K) - \operatorname{tr}\left(O_l\rho\right)| \leq \frac{\epsilon}{T} \quad \text{for all } 1 \leq l \leq L \tag{18}$$

with failure probability $\frac{\delta}{B}$.

Using Lemma 2 we can conclude that $K = \left\lceil 68\frac{T^2 3^k}{\epsilon^2}\log\left(\frac{2BL}{\delta}\right)\right\rceil$. Multiplying it by B gives us a total number of measurements. □

SUPPLEMENTARY NOTE 3 – PSEUDOCODE FOR THE TRAINING ALGORITHMS

Algorithm 1 *Joint*

Require: The gradient penalty coefficient λ, the number of critic parameters $\boldsymbol{w}$ updates per one model iteration $N_{\boldsymbol{w}}$, the batch size B, Adam hyperparameters for quantum circuit, observable and critic parameters $(\gamma^{\boldsymbol{\theta}}, \beta_1^{\boldsymbol{\theta}}, \beta_2^{\boldsymbol{\theta}}, \gamma^{\boldsymbol{\alpha}}, \beta_1^{\boldsymbol{\alpha}}, \beta_2^{\boldsymbol{\alpha}}, \gamma^{\boldsymbol{w}}, \beta_1^{\boldsymbol{w}}, \beta_2^{\boldsymbol{w}})$.
Require: Initial quantum circuit, observable, and critic parameters $(\boldsymbol{w}_0, \boldsymbol{\theta}_0, \boldsymbol{\alpha}_0)$.

```
while (θ, α) has not converged do
    for t = 1, ..., N_w do
        for i = 1, ..., B do
            Sample real data x^(i) ~ Q, latent variable z^(i) ~ P_z, a random number ε ~ U[0, 1].
            x̃^(i) ← G_{θ,α}(z^(i))
            x̂^(i) ← ε x^(i) + (1 − ε) x̃^(i)
        end for
        L_C ← (1/B) Σ_{i=1}^{B} [ D_w(x̃^(i)) − D_w(x^(i)) + λ ( ‖∇_x̂ D_w(x̂^(i))‖_2 − 1 )^2 ]
        w ← Adam(∇_w L_C, w, γ^w, β_1^w, β_2^w)
    end for
    Sample a batch of latent variables {z^(i)}_{i=1}^{B} ~ P_z
    L_G ← −(1/B) Σ_{i=1}^{B} D_w(G_{θ,α}(z^(i)))
    (θ, α) ← Adam(∇_{θ,α} L_G, (θ, α), (γ^θ, γ^α), (β_1^θ, β_1^α), (β_2^θ, β_2^α))
end while
```

Algorithm 2 *Asynchronous*

Require: The gradient penalty coefficient λ, the number of critic parameters $\boldsymbol{w}$ updates per one model iteration $N_{\boldsymbol{w}}$, the number of observable parameters $\boldsymbol{\alpha}$ updates per one model iteration $N_{\boldsymbol{\alpha}}$, the batch size B, Adam hyperparameters for quantum circuit, observable and critic parameters $(\gamma^{\boldsymbol{\theta}}, \beta_1^{\boldsymbol{\theta}}, \beta_2^{\boldsymbol{\theta}}, \gamma^{\boldsymbol{\alpha}}, \beta_1^{\boldsymbol{\alpha}}, \beta_2^{\boldsymbol{\alpha}}, \gamma^{\boldsymbol{w}}, \beta_1^{\boldsymbol{w}}, \beta_2^{\boldsymbol{w}})$.

Require: Initial quantum circuit, observable, and critic parameters $(\boldsymbol{w}_0, \boldsymbol{\theta}_0, \boldsymbol{\alpha}_0)$.

while $(\boldsymbol{\theta}, \boldsymbol{\alpha})$ has not converged **do**

  for $t = 1, \ldots, N_{\boldsymbol{w}}$ **do**

    for $i = 1, \ldots, B$ **do**

      Sample real data $\boldsymbol{x}^{(i)} \sim \mathbb{Q}$, latent variable $\boldsymbol{z}^{(i)} \sim \mathbb{P}_z$, a random number $\epsilon \sim U[0,1]$.

      $\tilde{\boldsymbol{x}}^{(i)} \leftarrow G_{\boldsymbol{\theta},\boldsymbol{\alpha}}(\boldsymbol{z}^{(i)})$

      $\hat{\boldsymbol{x}}^{(i)} \leftarrow \epsilon \boldsymbol{x}^{(i)} + (1-\epsilon)\tilde{\boldsymbol{x}}^{(i)}$

    end for

    $\mathcal{L}_C \leftarrow \frac{1}{B}\sum_{i=1}^{B}\left[D_{\boldsymbol{w}}(\tilde{\boldsymbol{x}}^{(i)}) - D_{\boldsymbol{w}}(\boldsymbol{x}^{(i)}) + \lambda\left(\left\|\nabla_{\hat{\boldsymbol{x}}} D_{\boldsymbol{w}}(\hat{\boldsymbol{x}}^{(i)})\right\|_2 - 1\right)^2\right]$

    $\boldsymbol{w} \leftarrow \mathrm{Adam}(\nabla_{\boldsymbol{w}}\mathcal{L}_C, \boldsymbol{w}, \gamma^{\boldsymbol{w}}, \beta_1^{\boldsymbol{w}}, \beta_2^{\boldsymbol{w}})$

  end for

  Sample a batch of latent variables $\{\boldsymbol{z}^{(i)}\}_{i=1}^{B} \sim \mathbb{P}_z$.

  for $j = 1, \ldots, N_{\boldsymbol{\alpha}}$ **do**

    $\mathcal{L}_G \leftarrow -\frac{1}{B}\sum_{i=1}^{B} D_{\boldsymbol{w}}(G_{\boldsymbol{\theta},\boldsymbol{\alpha}}(\boldsymbol{z}^{(i)}))$

    $\boldsymbol{\alpha} \leftarrow \mathrm{Adam}(\nabla_{\boldsymbol{\alpha}}\mathcal{L}_G, \boldsymbol{\alpha}, \gamma^{\boldsymbol{\alpha}}, \beta_1^{\boldsymbol{\alpha}}, \beta_2^{\boldsymbol{\alpha}})$

  end for

  $\mathcal{L}_G \leftarrow -\frac{1}{B}\sum_{i=1}^{B} D_{\boldsymbol{w}}(G_{\boldsymbol{\theta},\boldsymbol{\alpha}}(\boldsymbol{z}^{(i)}))$

  $\boldsymbol{\theta} \leftarrow \mathrm{Adam}(\nabla_{\boldsymbol{\theta}}\mathcal{L}_G, \boldsymbol{\theta}, \gamma^{\boldsymbol{\theta}}, \beta_1^{\boldsymbol{\theta}}, \beta_2^{\boldsymbol{\theta}})$

end while

Algorithm 3 *Decoupled*

Require: The gradient penalty coefficient λ, the number of critic parameters $\boldsymbol{w}$ updates per iteration $N_{\boldsymbol{w}}$, the number of observable parameters $\boldsymbol{\alpha}$ updates per iteration $N_{\boldsymbol{\alpha}}$, the batch size B, Adam hyperparameters for quantum circuit, observable and critic parameters $(\gamma^{\boldsymbol{\theta}}, \beta_1^{\boldsymbol{\theta}}, \beta_2^{\boldsymbol{\theta}}, \gamma^{\boldsymbol{\alpha}}, \beta_1^{\boldsymbol{\alpha}}, \beta_2^{\boldsymbol{\alpha}}, \gamma^{\boldsymbol{w}}, \beta_1^{\boldsymbol{w}}, \beta_2^{\boldsymbol{w}})$.

Require: Initial quantum circuit, observable and critic parameters $(\boldsymbol{w}_0, \boldsymbol{\theta}_0, \boldsymbol{\alpha}_0)$.

while $(\boldsymbol{\theta}, \boldsymbol{\alpha})$ has not converged **do**

  for $t = 1, \ldots, N_{\boldsymbol{\alpha}}$ **do**

    for $t = 1, \ldots, \lceil N_{\boldsymbol{w}}/N_{\boldsymbol{\alpha}} \rceil$ **do**

      for $i = 1, \ldots, B$ **do**

        Sample real data $\boldsymbol{x}^{(i)} \sim \mathbb{Q}$, latent variable $\boldsymbol{z}^{(i)} \sim \mathbb{P}_z$, a random number $\epsilon \sim U[0,1]$.

        $\tilde{\boldsymbol{x}}^{(i)} \leftarrow G_{\boldsymbol{\theta},\boldsymbol{\alpha}}(\boldsymbol{z}^{(i)})$

        $\hat{\boldsymbol{x}}^{(i)} \leftarrow \epsilon \boldsymbol{x}^{(i)} + (1-\epsilon)\tilde{\boldsymbol{x}}^{(i)}$

      end for

      $\mathcal{L}_C \leftarrow \frac{1}{B}\sum_{i=1}^{B}\left[D_{\boldsymbol{w}}(\tilde{\boldsymbol{x}}^{(i)}) - D_{\boldsymbol{w}}(\boldsymbol{x}^{(i)}) + \lambda\left(\left\|\nabla_{\hat{\boldsymbol{x}}} D_{\boldsymbol{w}}(\hat{\boldsymbol{x}}^{(i)})\right\|_2 - 1\right)^2\right]$

      $\boldsymbol{w} \leftarrow \mathrm{Adam}(\nabla_{\boldsymbol{w}}\mathcal{L}_C, \boldsymbol{w}, \gamma^{\boldsymbol{w}}, \beta_1^{\boldsymbol{w}}, \beta_2^{\boldsymbol{w}})$

    end for

    $\mathcal{L}_G \leftarrow -\frac{1}{B}\sum_{i=1}^{B} D_{\boldsymbol{w}}(G_{\boldsymbol{\theta},\boldsymbol{\alpha}}(\boldsymbol{z}))$

    $\boldsymbol{\alpha} \leftarrow \mathrm{Adam}(\nabla_{\boldsymbol{\alpha}}\mathcal{L}_G, \boldsymbol{\alpha}, \gamma^{\boldsymbol{\alpha}}, \beta_1, \beta_2^{\boldsymbol{\alpha}})$

  end for

  Sample a batch of latent variables $\{\boldsymbol{z}^{(i)}\}_{i=1}^{B} \sim \mathbb{P}_z$.

  $\mathcal{L}_G \leftarrow -\frac{1}{B}\sum_{i=1}^{B} D_{\boldsymbol{w}}(G_{\boldsymbol{\theta},\boldsymbol{\alpha}}(\boldsymbol{z}))$

  $\boldsymbol{\theta} \leftarrow \mathrm{Adam}(\nabla_{\boldsymbol{\theta}}\mathcal{L}_G, \boldsymbol{\theta}, \gamma^{\boldsymbol{\theta}}, \beta_1^{\boldsymbol{\theta}}, \beta_2^{\boldsymbol{\theta}})$

end while

SUPPLEMENTARY NOTE 4 – NUMERICAL EXPERIMENT SETUP

We use the Python package Tensorcircuit [3] for constructing quantum circuits, Equinox [4] for constructing the remaining architecture of the generator and the critic, `Jax` [5] for the simulation of training and sampling and `FAISS` [6] for the k-NN subroutine in the KLD estimator (Equation 25 of Ref. [7]). We perform all experiments on a single `NVIDIA RTX 2080 Ti GPU`.

This stack prioritizes the computational speed for simulating the training and sampling of OT-EVSs by making a copy of the quantum circuit for each observable, for each batch sample, and parallelizing all the circuits on the GPU. Our simulations, however, put a relatively high burden on GPU memory. The GPU memory consumption scales not only in the number of qubits n, the number of Pauli strings L, and the batch size B but also significantly in the number of quantum circuit parameters $\boldsymbol{\theta}$ because of the gradient storage in automatic differentiation. Simulations of

the shadow measurement scheme, for which the expectation values of $2k-$local observables need to be computed for variance approximation (Methods), consume more memory than those of the conventional measurement scheme by an asymptotic factor of n^k. Consequently, we only use conventional measurements for some of our numerical experiments with larger models.

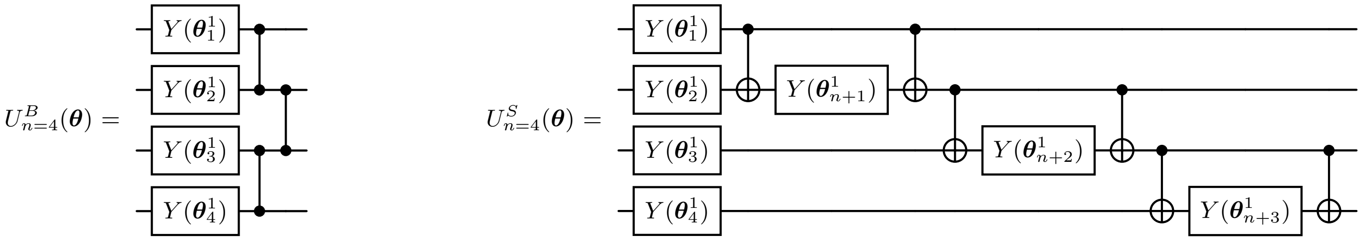

Supplementary Figure 1: The circuit diagrams for one layer of brickwork ansatz (left) and one layer of sequential ansatz (right) for 4 qubits.

We examined two circuit ansatze, *sequential* and *brickwork*, each with a different encoding circuit. The circuit diagrams for one layer of sequential ansatz (denoted by U^S) and one layer of brickwork ansatz (denoted by U^B) are shown in Figure 1. We consider different combinations of latent variable embedding strategies and variational ansatz. For the illustrative example in "Methods", we use

$$U^S \bigotimes_{j=1}^{n} X(z_2) U^S \bigotimes_{j=1}^{n} X(z_1).$$

For the sequential and brickwork circuits in "Results", we use

$$\left(U^S\right)^{N_l} \bigotimes_{j=1}^{n} Z_j(z_2) \bigotimes_{j=1}^{n} X_j(z_1) \quad \text{and} \quad \left(U^B\right)^{N_l} \bigotimes_{\substack{j=1 \\ j \text{ odd}}}^{n} X_j(z_1) \bigotimes_{\substack{k=1 \\ k \text{ even}}}^{n} X_k(z_2).$$

In the numerical experiments for training methods and shot noise, we examined models with both ansätze across three configurations (number of qubits n, number of circuit layers N_l, locality of observables k, data dimension M):

$$\text{(C1)}\ \ n = 8,\ N_l = 2,\ k = 1,\ M = 8,$$

$$\text{(C2)}\ \ n = 8,\ N_l = 9,\ k = 1,\ M = 8,$$

$$\text{(C3)}\ \ n = 11,\ N_l = 2,\ k = 2,\ M = 64.$$

In all numerical experiments, the models are initialized as follows before training: The quantum circuit parameters are drawn from the uniform distribution on $[-\pi, \pi)$, and the observable and critic parameters are set according to the Kaiming Initialisation [8]. We always use the hyperparmeters $N_{\boldsymbol{w}} = N_{\boldsymbol{\alpha}} = 5$ for training. The other hyperparameters for training are searched based on a randomized grid search, with results summarized in Table I. For a fair comparison, we use exactly the same hyperparameter setting for all experiment cases, i.e., across all three training algorithms, measurement schemes, and all model configurations for the same training algorithm and measurement scheme. We evaluate the model performance (KL divergence) using 2048 generated samples (maximum allowed by FAISS [6]) and 2048 training data.

We train autoencoders to downscale the MNIST and Fashion-MNIST datasets. The two autoencoders have the same architecture, except that the data are downscaled to 8 and 16 dimensions, respectively. The encoder is a multi-layer perceptron of two hidden layers (128 and 64 neurons) with RELU activation function. The decoder has the reversed architecture with an additional final sigmoid function.

Supplementary Table I: List of hyperparameters used in numerical experiments. We perform hyperparameter optimization by random grid search for each experiment. All trials in each experiment (for all WGAN variants, all measurement schemes, or all circuit depths, when applicable) share the same hyperparameters.

		WGAN Hyperparameters												
		λ	$N_{\boldsymbol{w}}$	$N_{\boldsymbol{\alpha}}$	B	$\gamma^{\boldsymbol{\theta}}$	$\beta_1^{\boldsymbol{\theta}}$	$\beta_2^{\boldsymbol{\theta}}$	$\gamma^{\boldsymbol{\alpha}}$	$\beta_1^{\boldsymbol{\alpha}}$	$\beta_2^{\boldsymbol{\alpha}}$	$\gamma^{\boldsymbol{w}}$	$\beta_1^{\boldsymbol{w}}$	$\beta_2^{\boldsymbol{w}}$
Illustration ($n = 4$)		0.1	5	5	256	10^{-3}	0	0.9	10^{-4}	0.9	0.9	10^{-4}	0.5	0.9
Compare Training Settings	Seq.(C1)	0.1	5	5	256	10^{-3}	0	0.99	10^{-4}	0	0.9	10^{-4}	0.9	0.99
	Seq.(C2)	0.1	5	5	256	10^{-3}	0	0.5	10^{-4}	0	0.9	10^{-4}	0.5	0.9
	Seq.(C3)	0.1	5	5	256	10^{-2}	0.5	0.5	10^{-4}	0.5	0.9	10^{-4}	0.5	0.9
	Brk.(C1)	0.1	5	5	256	10^{-3}	0	0.9	10^{-4}	0	0.99	10^{-4}	0	0.99
	Brk.(C2)	0.1	5	5	256	10^{-3}	0	0.5	10^{-4}	0	0.9	10^{-4}	0.5	0.9
	Brk.(C3)	0.1	5	5	256	10^{-2}	0.5	0.5	10^{-4}	0	0.5	10^{-4}	0.5	0.9
OT- vs. OF-EVS	Seq.	0.1	5	5	256	10^{-3}	0	0.5	10^{-4}	0	0.9	10^{-4}	0.5	0.9
	Brk.	0.1	5	5	256	10^{-3}	0	0.5	10^{-4}	0	0.9	10^{-4}	0.5	0.9
Image Datasets	MNIST	1	5	5	256	10^{-2}	0.9	0.9	10^{-3}	0	0.99	10^{-3}	0.5	0.5
	Fashion	1	5	5	256	10^{-2}	0.9	0.9	10^{-3}	0	0.99	10^{-3}	0.5	0.5

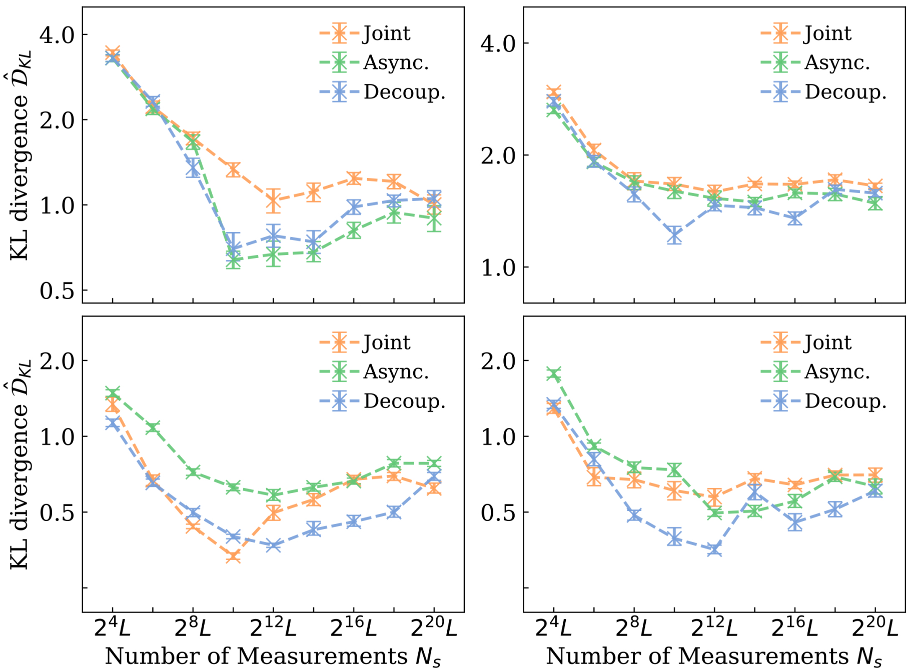

Supplementary Figure 2: Training performance of OT-EVS with (a) a nine-layer 8-qubit *sequential* circuit (b) a nine-layer 8-qubit *brickwork* circuit (c) a two-layer 11-qubit *sequential* circuit (d) a two-layer 11-qubit *brickwork* circuit using conventional measurements on the synthetic dataset. The interquartile mean and bootstraped 95% confidence intervals over 20 trials for the estimated KL divergence after $50k$ training iterations are shown.

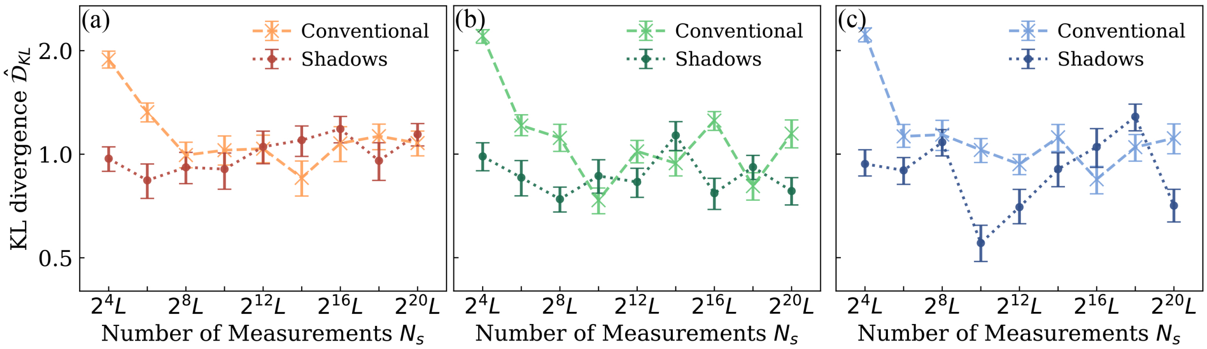

Supplementary Figure 3: Training performance of OT-EVS with a two-layer 8-qubit *brickwork* circuit using the (a) *Joint* (b) *Asynchronous* (c) *Decoupled* method on the synthetic dataset. The interquartile mean and bootstraped 95% confidence intervals over 20 trials for the estimated KL divergence after $50k$ training iterations are shown.

[1] X. Bonet-Monroig, H. Wang, and A. Pérez-Salinas, Verifying randomness in sets of quantum states via observables (2024), arXiv:2404.16211 [quant-ph].

[2] H.-Y. Huang, R. Kueng, and J. Preskill, Predicting Many Properties of a Quantum System from Very Few Measurements, Nature Physics **16**, 1050 (2020), arXiv:2002.08953 [quant-ph].

[3] S.-X. Zhang, J. Allcock, Z.-Q. Wan, S. Liu, J. Sun, H. Yu, X.-H. Yang, J. Qiu, Z. Ye, Y.-Q. Chen, C.-K. Lee, Y.-C. Zheng, S.-K. Jian, H. Yao, C.-Y. Hsieh, and S. Zhang, TensorCircuit: a Quantum Software Framework for the NISQ Era, Quantum **7**, 912 (2023).

[4] P. Kidger and C. Garcia, Equinox: neural networks in JAX via callable PyTrees and filtered transformations (2021), arXiv:2111.00254.

[5] J. Bradhury, R. Frostig, P. Hawkins, and M. James Johnson, JAX: composable transformations of Python+NumPy programs (2018).

[6] M. Douze, A. Guzhva, C. Deng, J. Johnson, G. Szilvasy, P.-E. Mazaré, M. Lomeli, L. Hosseini, and H. Jégou, The Faiss library (2024), arXiv:2401.08281.

[7] Q. Wang, S. R. Kulkarni, and S. Verdu, Divergence Estimation for Multidimensional Densities Via k-Nearest-Neighbor Distances, IEEE Transactions on Information Theory **55**, 2392 (2009).

[8] K. He, X. Zhang, S. Ren, and J. Sun, Delving Deep into Rectifiers: Surpassing Human-Level Performance on ImageNet Classification, in *2015 IEEE International Conference on Computer Vision (ICCV)* (IEEE, Santiago, Chile, 2015) pp. 1026–1034.
